# Corn dried distillers grains with solubles (cDDGS) in the diet of pigs change the expression of adipose genes that are potential therapeutic targets in metabolic and cardiovascular diseases

**DOI:** 10.1186/s12864-018-5265-x

**Published:** 2018-12-03

**Authors:** Maria Oczkowicz, Tomasz Szmatoła, Małgorzata Świątkiewicz, Klaudia Pawlina-Tyszko, Artur Gurgul, Tomasz Ząbek

**Affiliations:** 1 Department of Molecular Biology of Animals, National Research Institute of Animal Production, ul Krakowska 1, 32-083 Balice Cracow, Poland; 2 Department of Nutrition Physiology, National Research Institute of Animal Production, Cracow, Poland

**Keywords:** Obesity, Pigs, cDDGS, RNA-seq, Backfat, Metabolic diseases, CVD, Alzheimer’s

## Abstract

**Background:**

Corn dried distillers grains with solubles (cDDGS) are a byproduct of biofuel and alcohol production. cDDGS have been used in pig feed for many years, because they are readily available and rich in protein, fiber, unsaturated fatty acids and phytosterols. However, feed mixtures too high in cDDGS result in the worsening of backfat quality. We performed RNA-sequencing analysis of backfat from crossbred pigs fed different diets. The diets were isoenergetic but contained different amounts of cDDGS and various sources of fats. The animals were divided into four dietary groups during the two months of experimentation: group I (control (-cDDGS+rapeseed oil)), group II (+cDDGS+rapeseed oil), group III (+cDDGS+beef tallow), and group IV (+cDDGS+coconut oil). The aim of the present experiment was to evaluate changes in the backfat transcriptome of pigs fed isoenergetic diets that differed in cDDGS presence.

**Results:**

Via DESeq2 software, we identified 93 differentially expressed genes (DEGs) between groups I and II, 13 between groups I and III, and 125 between groups I and IV. DEGs identified between group I (-cDDGS+rapeseed oil) and group II (+cDDGS+rapeseed oil) were highly overrepresented in several KEGG pathways: metabolic pathways (FDR < 1.21e-06), oxidative phosphorylation (FDR < 0.00189), fatty acid biosynthesis (FDR < 0.00577), Huntington’s disease (FDR < 0.00577), fatty acid metabolism (FDR < 0.0112), Parkinson’s disease (FDR < 0.0151), non-alcoholic fatty liver disease (NAFLD) (FDR < 0.016), Alzheimer’s disease (FDR < 0.0211) and complement and coagulation cascades (FDR < 0.02).

**Conclusions:**

We observed that the addition of cDDGS positively affects the expression of several genes that have been recently proposed as potential targets for the treatment of obesity, diabetes, cardiovascular disease, and Alzheimer’s disease (e.g.*, FASN, AACS, ALAS1, HMGCS1,* and *VSIG4*). Thus, our results support the idea of including cDDGS into the diets of companion animals and humans and encourage research into the bioactive ingredients of cDDGS.

**Electronic supplementary material:**

The online version of this article (10.1186/s12864-018-5265-x) contains supplementary material, which is available to authorized users.

## Background

The optimal feeding strategy for farm animals is one of the fundamental factors determining the profitability of meat production. cDDGS (corn dried distillers grains with solubles) has commonly been used as a replacement for soybean meal in animal feed due to its high protein and fiber content, reasonable price and availability. However, backfat from animals fed diets containing cDDGS is soft and prone to oxidation [1]. Fats containing high amounts of saturated fatty acids, such as beef tallow or coconut oil, are added to the feedstuff to counteract this deterioration in backfat quality. Such cDDGS have also been proposed for use in human diets, especially those of diabetic and celiac patients [1]. Interestingly, a decade ago, a beneficial effect of DDGS on ischemic heart disease was postulated and patented by the US Patent and Trademark Office (U.S. Patent No. 2004/0234630) [2]. Currently, with the increasing production of biofuels, the availability of cDDGS is high; however, its usage in human diets remains negligible. Understanding the molecular processes that occur in animal tissues after the consumption of cDDGS and different fats could resolve doubts surrounding the use of these compounds as a feedstuff and as part of companion animal and human nutrition.

To date, several experiments have revealed that nutrition may induce changes in the transcriptome and specific metabolic pathways [3]. Peñagaricano et al. (2014) observed noticeable gene expression differences in adipose tissue between sheep fetuses whose mothers were fed diets with cDDGS and those whose mothers were fed other isoenergetic diets during pregnancy [4]. Many of these genes were involved in adipogenesis, lipogenesis and adipose tissue development. We hypothesize that these observations may be relevant to other mammalian species. Therefore, we aimed to evaluate whether the addition of cDDGS to the diet of pigs changes the backfat transcriptome. Recently, pigs have become a promising alternative to rodent animal models due to similarities to humans with regard to organ size and physiology [5]; therefore, the results of our study may provide information concerning the possibility of using cDDGS as a health-promoting additive in the human diet.

The aims of the present study were to evaluate changes in the backfat transcriptome of pigs fed isoenergetic diets that differed in cDDGS content and sources of fat. Moreover, we intended to assess potential interactions between cDDGS and fatty acid compositions in the diet.

## Results

### The performance and subcutaneous backfat quality of animals after different diet treatments

Animal performance and backfat quality are described in detail elsewhere for the following dietary treatments: control - group I (-cDDGS+rapeseed oil), group II (+cDDGS+rapeseed oil), group III (+cDDGS+beef tallow), and group IV (+cDDGS+coconut oil) [6, 7]. In brief, the different dietary treatments did not affect weight gain, feed utilization, backfat thickness, or carcass meatiness. However, backfat from animals receiving cDDGS and rapeseed oil in their feed mixture (group II) displayed a lowest ratio of the sum of saturated fatty acids to the sum of unsaturated fatty acids (SFA:UFA), and the highest C18:2 linoleic acid content and iodine value (*p* < 0.001) (Table 1).

### RNA-seq statistics

After RNA-seq analysis, we obtained from 8,659,689 to 25,360,573 filtered reads per sample (Table 2). The percentage of mapped reads was between 79,5-84,4%. Up to 5,668,453 of reads were mapped uniquely to Ensembl *Sus scrofa* 11.1 with an annotation version 87. The number of identified genes with normalized counts > 1 was between 13,148–14,391 depending on sample.

### Differentially expressed genes (DEGs) identified by RNA-seq analysis

In the present study, we classified genes as differentially expressed when the fold change was > ± 1.3 and the adjusted *p*-value was < 0.05. Initially, we performed a comparison between the –cDDGS+rapeseed oil group (*n* = 7) and three other groups, namely, the +cDDGS+rapeseed oil (*n* = 6), +cDDGS+beef tallow (*n* = 5), and + cDDGS+coconut oil (n = 5) groups (3 analyses in total). The highest number of DEGs was identified when group I (-cDDGS+rapeseed oil) was compared to group IV (+cDDGS+coconut oil) and to group II (+cDDGS+rapeseed oil), which showed 125 DEGs with 32 overexpressed and 93 underexpressed in the -cDDGS+rapeseed oil group and 93 DEGs with 44 underexpressed and 49 overexpressed in the -cDDGS+rapeseed oil group, respectively. The number of DEGs was much lower in group I versus group III, which had 13 DEGs, 5 of which were underexpressed and 8 of which were overexpressed (Additional file 1: Table S1). Next, we combined all groups with cDDGS (group II, III and IV) and compared them to the group without cDDGS (group I). In this comparison, we identified 155 DEGs, with 59 upregulated and 96 downregulated, in the –cDDGS+rapeseed oil group (Additional file 1: Table S1). All expression data were submitted to the GEO database (accession number: GSE101433).

**Table 1:** Chosen performance and backfat quality parameters

	Group I (-cDDGS +rapeseed oil) *n* = 8	Group II (+cDDGS +rapeseed oil) *n* = 8	Group III (+cDDGS +beef tallow) *n* = 8	Group IV (+cDDGS +coconut oil) *n* = 8	SEM	*P* value
Fattening results:						
Average daily body weight gains (g)	986	959	958	991	11.72	0.663
Averag feed utilization (kg)	3.07	3.16	3.17	3.03	0.04	0.564
Fatty acid profile and lipid peroxidation indicators:						
C18:2	10.6 ^a^	13.5 ^c^	11.8 ^b^	10.2 ^a^	0.3	< 0.001
SFA^a^	46.5 ^b^	43.5 ^a^	47.2 ^b^	52.3 ^c^	0.7	< 0.001
UFA^a^	52.4 ^b^	55.5 ^c^	51.7 ^b^	46.5 ^a^	0.7	< 0.001
MUFA^a^	39.8 ^b^	40.1 ^b^	38.6 ^b^	35.3 ^a^	0.5	0.001
PUFA n-6^a^	10.8 ^a^	13.8 ^c^	12.2 ^b^	10.4 ^a^	0.3	< 0.001
PUFA n-3^a^	1.76 ^c^	1.61 ^c^	0.98 ^b^	0.77 ^a^	0.09	< 0.001
PUFA^a^	12.6 ^b^	15.4 ^c^	13.2 ^b^	11.2 ^a^	0.3	< 0.001
SFA/UFA ratio	0.89 ^b^	0.78 ^a^	0.91 ^b^	1.12 ^c^	0.03	< 0.001
IV (g/100 g)^b^	57.2 ^b^	62.0 ^c^	56.3 ^b^	50.0 ^a^	0.9	< 0.001
TBA-RS (mg/kg)^c^	0.25 ^ab^	0.30 ^b^	0.25 ^ab^	0.18 ^a^	0.02	0.029

^a.b.c^. Values within a row with different superscripts differ significantly at *P* < 0.05

^a^Sum of fatty acids: saturated (SFA). unsaturated (UFA). monounsaturated (MUFA). polyunsaturated (PUFA)

^b^IV – iodine value of fat

^c^TBA-RS - Thiobarbituric acid reactive substances

**Table 2:** RNA-seq statistics

Samples	Statistics of reads			
	Number of filtered reads	Number of mapped reads	Percentage of mapped reads (%)	Number of reads uniquely mapped to genes^a^
1	16,937,563	14,063,133	83.0	4,898,483
2	15,611,798	12,818,778	82.1	5,261,962
3	14,021,444	11,828,328	84.4	4,530,749
5	16,623,142	13,027,265	78.4	5,085,954
6	10,284,981	8,369,135	81.4	3,006,222
7	14,243,552	11,732,846	82.4	4,243,290
8	15,773,245	12,596,918	79.9	4,922,991
17	14,394,849	11,897,291	82.6	4,717,511
19	13,255,853	10,795,981	81.4	3,005,356
20	17,022,708	14,146,517	83.1	5,123,759
21	13,860,471	11,408,161	82.3	3,142,708
22	14,599,238	11,862,460	81.3	3,747,760
24	8,659,689	6,888,176	79.5	2,437,172
41	13,056,710	10,918,790	83.6	3,768,842
43	19,788,884	16,497,403	83.4	5,668,453
45	14,887,931	12,403,405	83.3	4,848,317
46	25,360,573	20,844,592	82.2	7,979,426
48	14,089,646	11,599,227	82.3	5,033,537
49	17,957,567	14,466,678	80.6	4,826,659
52	13,823,894	11,246,208	81.4	3,762,672
53	16,045,183	13,208,046	82.3	4,865,260
54	17,361,291	14,101,605	81.2	3,697,673
55	14,380,150	11,892,987	82.7	4,079,125

^a^in regard to Ensembl SusScrofa 11.1

**Fig. 1:**
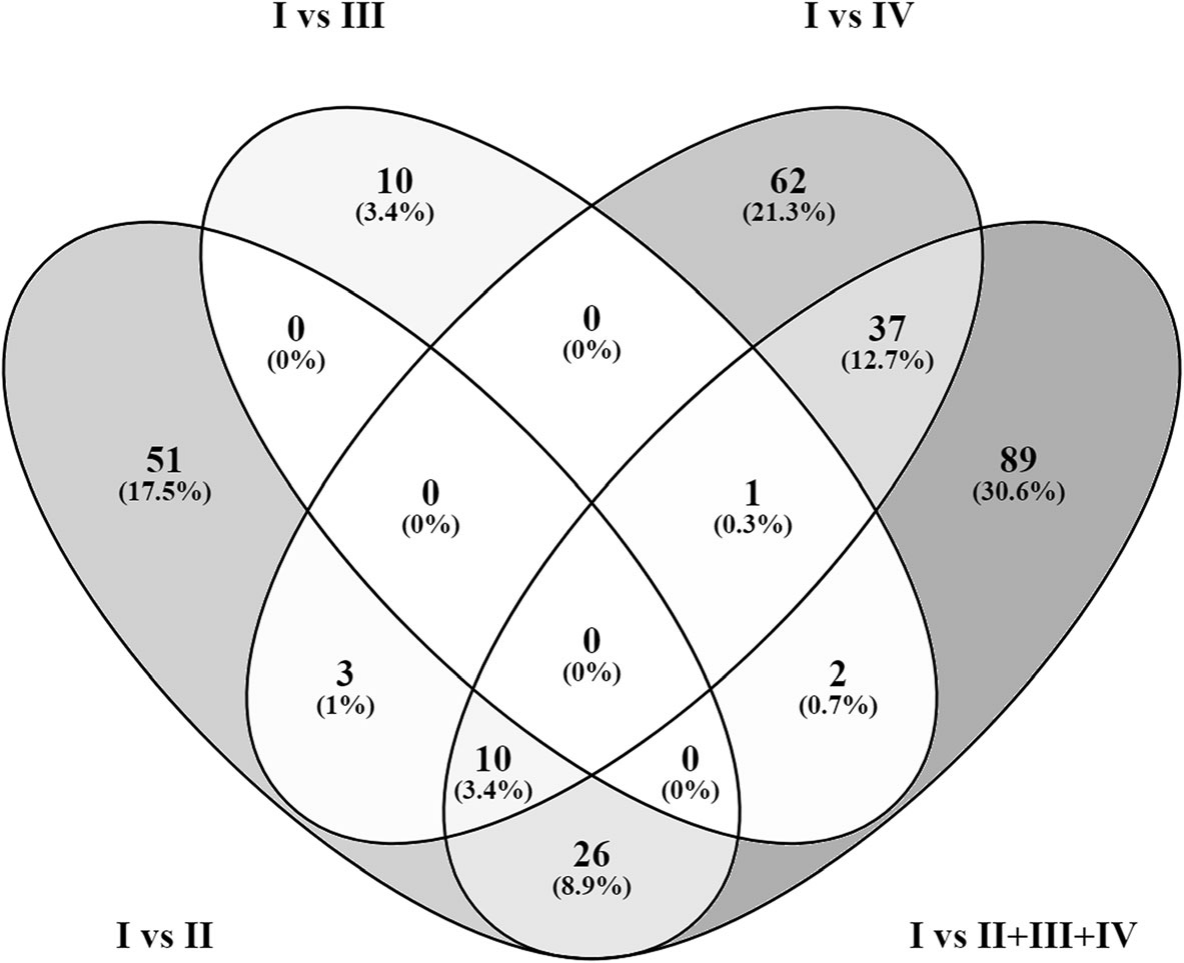
The Venn diagram shows common DEGs among four comparisons: (group I vs group II), (group I vs group III), (group I vs group IV) and (group I vs II + III + IV)

The comparison of DEG lists with the Venny integrative tool (http://bioinfogp.cnb.csic.es/tools/venny/index.html) revealed 39 (13,3%) common genes between (group I vs group II) and (group I vs II + III + IV), 48 (16,4%) common genes between (group I vs group IV) and (group I vs group II + III + IV) and only 3 (1%) common genes between (group I vs group III) and (group I vs group II + III + IV) (Fig. 1).

### Functional analysis of identified DEGs

To draw proper conclusions about the effect of each dietary factor on biological processes, molecular functions and pathways we analyzed each dataset of DEGs separately and did not analyze DEGs from (group I vs group II + III + IV) comparison. We performed several analyses to identify overrepresented DEGs using the PANTHER classification system. The most interesting results were obtained when the DEGs overexpressed in group I (-cDDGS+rapeseed oil) were compared to those in group II (+cDDGS+rapeseed oil) (47 mapped genes of the 49 DEGs). The DEGs were mainly involved in the following pathways: metabolic processes, fatty acid biosynthetic process, lipid metabolic process, and coenzyme metabolic process (Table 3). Many of these genes encoded enzymes that catalyze metabolic processes; therefore, catalytic activity and ligase activity were overrepresented among the molecular functions. Overexpressed genes were overrepresented in several Reactome pathways, of which the most significant was ChREBP, which activates metabolic gene expression (FDR < 3.94E-06), metabolism (FDR < 1.51E-10), metabolism of lipids and lipoproteins (FDR < 3.10E-05), fatty acyl-CoA biosynthesis (FDR < 4.57E-05), the citric acid (TCA) cycle and respiratory electron transport (FDR < 1.61E-02) (Table 3). No significant enrichments were observed for DEGs in the group I vs group III or the group I vs group IV comparisons.

**Fig. 2:**
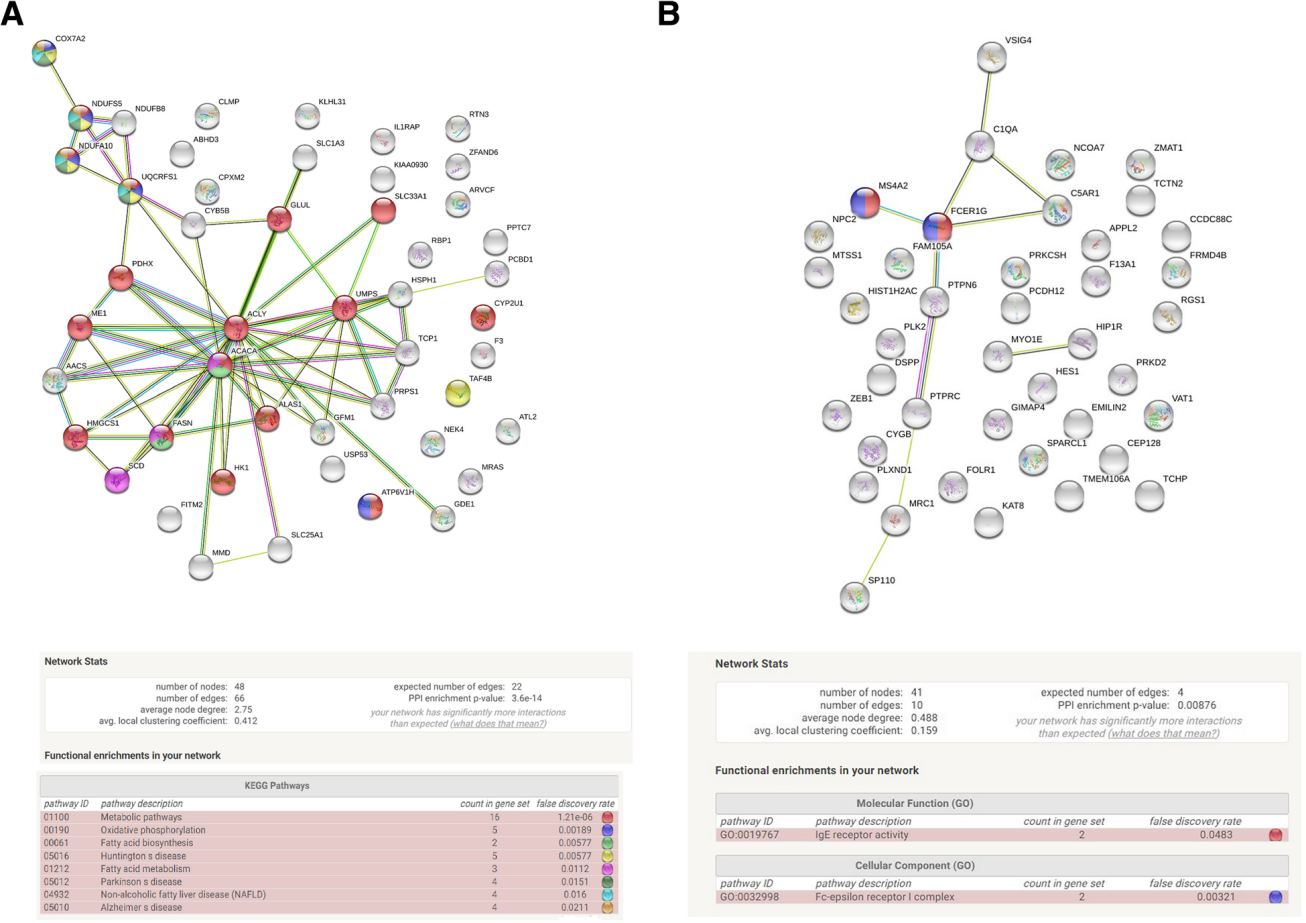
Network of interacting proteins obtained after String 10.0 bioinformatics software analysis (http://string-db.org) of DEGs in (–cDDGS+rapeseed oil) vs (+cDDGS+rapeseed oil) comparison. **a** DEGs downregulated in (+cDDGS+rapeseed oil), **b** DEGs upregulated in (+cDDGS+rapeseed oil genes)

Next, we employed STRING Software (https://string-db.org) to find networks of DEGs identified in our experiment. The network of DEGs identified in group I (–cDDGS+rapeseed oil) vs group II (+cDDGS+rapeseed oil) had significantly more interactions than expected (PPI enrichment *p* < 1.01E-06), while for all other comparisons, this *p*-value was greater than 0.05, suggesting the absence of strong interactions. However, we found that in the comparison of group I vs group IV, genes of tight junction proteins (FDR < 0.016) as well as genes of organelles and vesicles (FDR < 0.0012, FDR < 0.014) were overrepresented. We performed three analyses of the network of genes from the group I vs group II comparison: an analysis of all differentially expressed genes (90 mapped genes of 93 DEGs), an analysis of downregulated genes (48 mapped genes of 49 DEGs) (Fig. 2a), and an analysis of upregulated genes (42 mapped genes of 44 DEGs) (Fig. 2b). In all these analyses, we observed highly significant networks (p < 1.01E-06, *p* < 3.6E-14, *p* < 0.000975, respectively). The most overrepresented pathways were metabolic pathways, fatty acid biosynthesis, oxidative phosphorylation, and complement and coagulation cascades. A list of all functional enrichments is presented in Additional file 2: Table S2.

To further analyze the function of genes regulated by cDDGS, we proposed a set of high-confidence genes that were differentially expressed in both DESeq2 analyses (group I vs group II and group I vs group II + III + IV) (Table 4) and noticed that according to the literature, many are potential therapeutic targets in obesity, diabetes, and cardiovascular and neurodegenerative diseases.

### Validation of RNA-seq analysis by qPCR

**Fig. 3:**
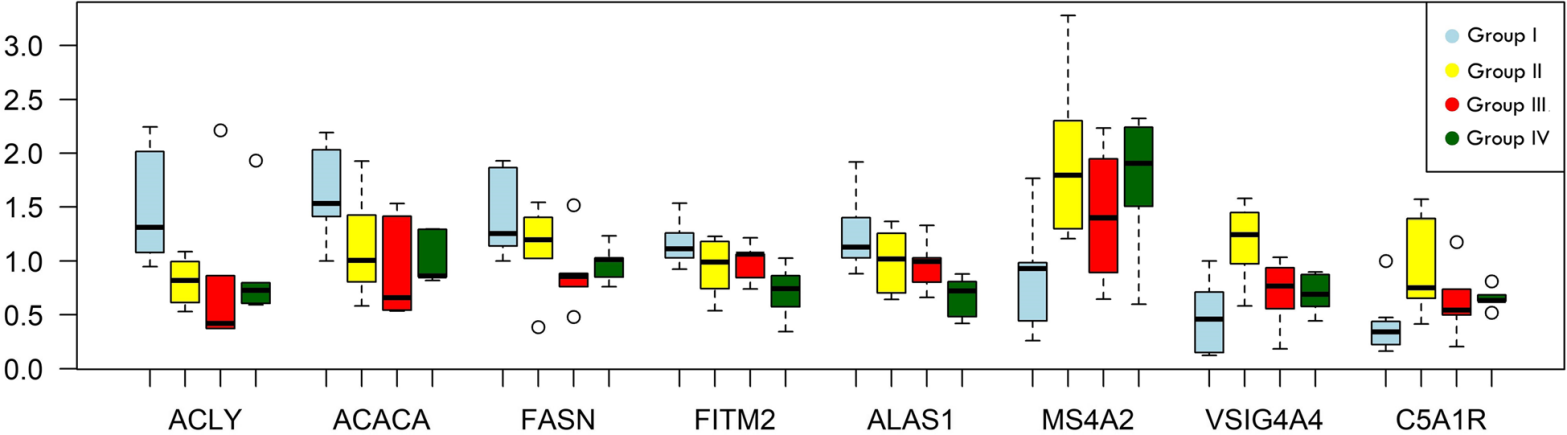
Results of qPCR analysis of selected genes in each dietary groups: group I (-cDDGS+rapeseed oil), group II (+cDDGS+rapeseed oil), group III (+cDDGS+beef tallow), group IV (+cDDGS+coconut oil) after GLM procedure with diet and sex as fixed factors. *p-value < 0.05, ** f3:2 *p*-value < 0.01, ns - *p* > 0.1

We performed qPCR analysis of eight genes, five of which were downregulated *(ACACA, ACLY, FASN, FITM2,* and *ALAS)* and three of which were upregulated *(VSIG4, C5AR1,* and *MS4A2)* in group II (+cDDGS+rapeseed oil) (Additional file 1: Table S1). The results of qPCR analysis for each dietary group are presented in Fig. 3. Statistical analysis with GLM procedure revealed significance of diet for *ACACA, FITM2, ALAS1, MS4A2* and *VSIG4* expression, while for *ACLY, FASN* and *C5AR1* trends were observed (*p* < 0.074, 0.063, 0.058 respectively). Sex was a significant factor only for *VSIG4* gene expression (Fig. 3). To compare fold changes obtained by RNA-seq and qPCR we used analysis with combined groups (group I) vs (group II + II + IV)(Additional file 3: Figure S1). All expression patterns obtained by qPCR were in the same direction as the patterns obtained by RNA-seq (Additional file 3: Figure S1). The Pearson correlation (R^2^ = 0.99) between the qPCR and RNA-seq results was highly significant, at *p* < 0000001.

## Discussion

Using cDDGS in the human diet was proposed more than a decade ago due to its beneficial impact on cardiovascular diseases and possible use as a low-cost flavor enhancer and sodium reduction enabler (US Patents: 2004/0234630 and US20080160132A1). Recently, the idea of including DDGS as an ingredient in food has been reconsidered [8, 9]. Moreover, a new method for the mitigation of mycotoxins, which are the main hazardous contaminant of cDDGS, has been developed, increasing the safety of this byproduct [10]. Nevertheless, the putative molecular mechanisms by which cDDGS prevent cardiovascular diseases have not been identified to date. Several experiments conducted to investigate gene expression changes after the inclusion of cDDGS in the diet of animals [11–14] revealed that cDDGS influence the expression of nutrient receptor genes as well as immune-associated and lipid metabolic processes. It was shown, for example, that a diet based on cDDGS increased the number of goblet cells and the intestinal expression of the *MUC2, PEPT1* and *CASR* genes in pigs. The *PEPT1* and *CASR* genes are responsible, for the immune response and barrier function in piglets [11], an observation that corresponds well with our results demonstrating the upregulation of genes connected to immunity in adipose tissue. Interestingly, the authors also noted a reduction in the intestinal expression of the umami taste receptor *TAS1R3*, which has been suggested to precipitate in metabolic regulation. Our results show that cDDGS significantly change the expression of genes associated with metabolism (FDR < 1.51E-10) (Table 3) in adipose tissue. It is tempting to speculate that these changes may be the result of processes occurring in the gut after cDDGS consumption. Moreover, many metabolism-related genes have previously been reported as potential therapeutic targets in metabolic disorders as they are associated with several putative common pathomechanisms suggested in obesity, diabetes, cancer, cardiovascular and neurodegenerative diseases (Table 4).

**Table 3:** Panther Classification System analysis of enrichment of Biological processes. Molecular functions and reactome pathways among overexpressed genes in the animals fed control diet (-DDGS + rapeseed oil) when compared to animals fed (+DDGS+rapeseed oil) diet (FDR) - False Discovery Rate

	FDR
PANTHER GO-Slim Biological Process	
fatty acid biosynthetic process	2.61E-02
lipid metabolic process	4.87E-04
metabolic process	3.93E-02
coenzyme metabolic process	4.38E-04
PANTHER GO-Slim Molecular Function	
ligase activity	1.27E-05
catalytic activity	1.26E-02
Reactome pathways	
ChREBP activates metabolic gene expression	3.94E-06
Integration of energy metabolism	2.47E-02
Metabolism	1.51E-10
Activation of gene expression by SREBF (SREBP)	2.34E-02
Regulation of cholesterol biosynthesis by SREBP (SREBF)	4.18E-02
Metabolism of lipids and lipoproteins	3.10E-05
Fatty Acyl-CoA Biosynthesis	4.57E-05
Triglyceride Biosynthesis	3.36E-04
Fatty acid. Triacylglycerol. and ketone body metabolism	4.58E-04
Respiratory electron transport	3.04E-02
The citric acid (TCA) cycle and respiratory electron transport	1.61E-02

**Table 4:** Common genes differentially expressed in two comparisons: group I vs group II and group I vs group II + III + IV, related to human disease: NAFLD-Non Alcoholic Fatty Liver Disease, NASH –Non-Alcoholic SteatoHepatitis, OB-obesity, CAD-Cardiovascular Disease, T1D – diabetes Mellitus type I, T2DM – Diabetes Melitus type II, ND-Neurodegenarative Disease, ID – Immunity Disease

Regulation by cDDGS	official gene symbol	official gene name	Disease
up	*KAT8*	lysine acetyltransferase 8	NAFLD, NASH, cancer [43, 44]
up	*CYGB*	cytoglobin	OB,CAD, cancer [45, 46]
up	*GIMAP4*	GTPase, IMAP family member 4	T1D, ID [47]
up	*HIP1R*	huntingtin interacting protein 1 related	ND [48]
up	*CEP128*	centrosomal protein 128	
up	*FAM105A*	family with sequence similarity 105, member A	T2D [49]
up	*PCDH12*	protocadherin 12	CAD [50]
up	*C5AR1*	complement component 5a receptor 1	OB, ND, CAD [41, 42, 51]
up	*ZMAT1*	zinc finger matrin-type 1	
up	*VSIG4*	V-set and immunoglobulin domain containing 4	OB, ID [52]
up	*SP110*	SP110 nuclear body protein	ID [53]
up	*SPARCL1*	SPARC like 1	OB, CAD, ND, cancer [54–56]
up	*TCHP*	trichoplein keratin filament binding	
up	*DSPP*	dentin sialophosphoprotein	
up	*MS4A2*	membrane spanning 4-domains A2	ND, allergy
up	*MYO1E*	myosin IE	ND [57]
up	*NCOA7*	nuclear receptor coactivator 7	
up	*RGS1*	regulator of G protein signaling 1	CAD [58]
up	*ZEB1*	zinc finger E-box binding homeobox 1	OB, CAD [17, 18]
down	*FASN*	fatty acid synthase	OB, T2D, NAFLD, cancer [19, 20, 59]
down	*SLC25A1*	solute carrier family 25 member 1	cancer, T2D, ND [60]
down	*HMGCS1*	3-hydroxy-3-methylglutaryl-CoA synthase 1	OB, T2D, CAD [61]
down	*ALAS1*	5′-aminolevulinate synthase 1	OB, NAFLD, CAD, T2D, ND [62, 63]
down	*RTN3*	reticulon 3	T2D,CAD, ND [64, 65]
down	*AACS*	acetoacetyl-CoA synthetase	NAFLD, ND [66, 67]
down	*ARVCF*	ARVCF, delta catenin family member	CAD [68]
down	*UMPS*	uridine monophosphate synthetase	OB, cancer [69–71]
down	*NDUFB8*	NADH:ubiquinone oxidoreductase subunit B8	
down	*TAF4B*	TATA-box binding protein associated factor 4b	
down	*GFM1*	G elongation factor mitochondrial 1	
down	*NDUFS5*	NADH:ubiquinone oxidoreductase subunit S5	
down	*PDHX*	pyruvate dehydrogenase complex component X	T2D, ND [72]
down	*CYP2U1*	cytochrome P450 family 2 subfamily U member 1	
down	*FITM2*	fat storage-inducing transmembrane protein 2	OB. [73]

In our experiment, we observed the highest number of DDGS in two comparisons: -DDGS+rapeseed oil vs + cDDGS +rapeseed oil and -cDDGS +rapeseed oil vs + cDDGS+coconut oil (Additional file 1: Table S1). However, only the genes from the first comparison were highly connected to each other, as only this dataset created a significant network of interactions in STRING analysis (Fig. 3). The reason for the lack of significant interactions in group I vs group III and group I vs group IV may be the presence of two different dietary factors (cDDGS and fat), which could make the gene dataset too heterogeneous. We performed additional analysis of -cDDGS vs + cDDGS by combining all three groups with cDDGS to take advantage of a larger number of samples and to verify how many DEGs would still be significant with the additional dietary factors (beef tallow and coconut oil). Interestingly, many of the DEGs common in both analyses are connected to metabolic diseases and are considered potential therapeutic targets (e.g.*, FASN, RTN3)* (Table 4). In contrast, the comparison of group I with group III led us to identify only 13 DEGs, which suggests that cDDGS and beef tallow may have an opposite effect on these biological pathways. Previously, it was suggested that dietary fat and fiber have antagonistic effects on adipose inflammation [15]. In our experiment, the diets with cDDGS had only 3.2–4.7% more fat than the diets without cDDGS, but the diets differed considerably in the SFA:UFA ratio and PUFA n-6 content (Table 1), which suggests that the source and fatty acid composition of dietary fat may also be important in modulating metabolic and inflammatory pathways. The comparison of -cDDGS+rapessed oil with +cDDGS+coconut oil resulted in the identification of 125 DEGs mainly engaged in metabolic processes and catalytic activity (data not shown), suggesting that coconut oil and cDDGS may have a synergistic effect on some metabolism-related genes. Nonetheless, the genes identified in this comparison did not create significant networks. In addition, there was a strong prevalence of overexpressed to underexpressed genes in the +cDDGS+coconut oil group. Therefore, it would be interesting to further investigate the transcriptomic effects of the combination of cDDGS and coconut oil.

### Fatty acid biosynthesis is downregulated by cDDGS

Several genes involved in fatty acid biosynthesis were downregulated by the presence of cDDGS in the diet (*FASN, ACLY,* and *SCD) (*Additional file 1: Table S1, Additional file 2: Table S2) (Fig. 3). A key enzyme of fatty acid biosynthesis, *FASN,* was downregulated when group I was compared to group II and to the combined three +cDDGS groups. In the other comparisons, (group I vs group IV and group I vs group III), the differences were not statistically significant for RNA-seq, but qPCR analysis suggested that all three groups with cDDGS had decreased expression of *FASN* (Fig. 3). The same result was noted for the *ACLY, FITM2 and ACACA* genes. Simultaneously, *KAT8,* a gene responsible for the acetylation and degradation of *FASN* [16], and *ZEB1*, a transcription factor which is a well-known adipogenesis repressor [17, 18], were upregulated in group II and in the combined groups with cDDGS (II + III + IV) when compared to group I. Recently, it has been proposed that *FASN* inhibitors have therapeutic utility in diseases associated with elevated lipogenesis, such as obesity, type 2 diabetes, and NAFLD. The first FASN inhibitor successfully advanced through the drug development process and is under clinical evaluation in oncology [19]. Moreover, it was shown that certain anti-diabetic foods, like baker’s yeast glucan (BYG) or tartary buckwheat extracts, downregulate genes responsible for fatty acid biosynthesis *(ACLY, ACC,* and *FASN.)* [19–22]. Our results show that cDDGS may have similar properties. In fact, as a byproduct of alcohol production, cDDGS contains a substantial amount of yeast debris [13]. It also contains high amounts of polyunsaturated fatty acids, which are well-known inhibitors of lipogenesis through the downregulation of *SCD* and *FASN* [23, 24]. In addition, cDDGS contain high amounts of fiber, which has strong anti-lipogenic activity [9]. A significant decrease in lipogenic gene expression (*ACACA, ACLY, FASN,* and *SCD*) in chickens and rats fed a fiber-enriched diet has been recently demonstrated [25, 26]. The potential mechanism by which dietary fiber decreases lipogenesis may be connected to altered gut microbiota and is currently being intensively investigated [27].

### Oxidative phosphorylation is downregulated by cDDGS

The second crucial process downregulated by cDDGS and connected to metabolic diseases was oxidative phosphorylation (Fig. 3b and Additional file 2: Table S2). In our study, we observed the upregulation of mitochondrial genes in animals fed a standard diet with no cDDGS. In particular, genes (*NDUFS5, NDUFA10, NDUFB8, COX7A2,* and *UQCRFS1*), *(COX8A)* and *(NDUFA10, NDUFB8, COX1,* and *UQCRQ)* were upregulated when group I was compared to groups II, IV and (II + III + IV), respectively (Additional file 1: Table S1 and Fig. 2a). Since the standard diet for pigs may be considered obesity-inducing, we speculate that the addition of cDDGS prevents diet-induced mitochondrial hyperactivation and possible mitochondrial dysfunction in the long term. In obesity and neurodegenerative diseases, impaired mitochondrial activity and biogenesis were observed [28, 29]. It has been recently demonstrated that mitochondrial alterations in the adipose tissue of mice are biphasic after diet-induced oxidative stress [30]. After two months of a high fat - high sucrose diet, mitochondrial biogenesis increased, while after 12 months, a substantial decrease was observed, accompanied by mitochondrial ultrastructure changes. Similarly, it was shown that in humans, yeast, worms, flies, mice, and monkeys that middle-age is accompanied by increased mitochondrial activity that subsequently declines at advanced ages [31]. A decrease in mitochondrial activity has been proposed as an anti-senescence strategy, since mitochondrial dysfunction and a hyperactive TCA cycle are important contributors to senescence [32, 31]. Interestingly, it has been recently shown that the *KAT8* gene, which was upregulated by the addition of cDDGS in our experiment, regulates oxidative phosphorylation by controlling the expression of respiratory genes [33]. This finding suggests that deregulation of this gene may be responsible for the alterations in respiratory electron transport and fatty acid biosynthesis observed in our study.

There were several downregulated genes (*ALAS1, CYB5B, PDHX,* and *ACLY*) (Fig. 3b) that were linked to both of the previously mentioned processes: fatty acid metabolism and cellular respiration. *ACLY* catalyzes the synthesis of acetylcoenzyme A, which is the main substrate in fatty acid biosynthesis as well as in the TCA cycle, while *ALAS 1* catalyzes heme biosynthesis for cytochrome P450, the terminal oxidase enzyme in the electron transfer chain. It has been recently proposed that heme biosynthesis is linked to adipogenesis through mitochondrial respiratory activity [34]. Researchers hypothesized that heme biosynthesis is required to achieve optimal adipocyte differentiation by sustaining mitochondrial function.

### Complement and coagulation cascade are upregulated by cDDGS

Previous studies indicate that cDDGS may have a stimulatory effect on the immunity of animals [12–14, 35]. The results of our experiment support this hypothesis since we observed that cDDGS significantly affect adipose expression of genes connected to immunity. STRING analysis of DEGs in group I (-cDDGS+rapeseed oil) vs group II (+cDDGS+rapeseed oil) shows that the addition of cDDGS results in differential expression of complement and coagulation cascade genes *(C1Q, C5AR1, F3,* and *F13A1)* (Fig. 3a) as well as IgE receptor activity and Fc epsilon receptor complex genes (*FCER1G* and *MS4A2)* (Additional file 2: Table S2). These genes (except for *FCER1G)* were also downregulated in the group with no cDDGS in the diet (group I) compared to the combined +cDDGS groups (II + III + IV) (Additional file 1: Table S1). Two additional genes related to the complement system *(C1QTNF6* and *C1QTNF9)* were differentially expressed in group I compared to the(+cDDGS +coconut oil) and (+cDDGS +beef tallow groups), respectively. Activation of complement cascades enhances the removal of bacteria and apoptotic cells and regulates inflammation. Both excessive stimulation and inhibition of this system negatively affect organisms. Chronic low-grade inflammation is considered one of the main factors contributing to the pathogenesis of obesity and neurodegenerative and cardiovascular diseases. Inflammation is initiated by polarization of M0 macrophages into pro-inflammatory M1 macrophages, while polarization to M2 macrophages has anti-inflammatory effects and is responsible for the phagocytosis of apoptotic cells. Recently, it has been shown that the *VSIG4* gene, which was one of the most upregulated genes by cDDGS in our study, (Additional file 1: Table S1) inhibits macrophage-mediated inflammation, high-fat diet-induced obesity, insulin resistance, severe fibrinogen formation in the liver, mitochondrial oxidation and ROS formation [36]. Researchers demonstrated that *VSIG4* reprograms mitochondrial pyruvate metabolism by increasing PDK2 expression in macrophages and the phosphorylation of pyruvate dehydrogenase (PDH) complex PDH-E. In our study, we observed that the upregulation of the *VSIG4* gene in the +cDDGS groups (Additional file 1: Table S1) was accompanied by the downregulation of subunit E3 of the pyruvate dehydrogenase-PDHX complex component X, suggesting that not only the phosphorylation of PDH but also the downregulation of the *PDHX*-E3 binding protein subunit of the pyruvate dehydrogenase complex may contribute to the decrease in PDH activity. In fact, mutation of the *PDHX* gene was shown to result in pyruvate dehydrogenase deficiency [37] in Moroccan patients, supporting our hypothesis.

There were several other anti-inflammatory murine markers among the genes upregulated by cDDGS (*MRC1* and *FOLR1*) [38] (Additional file 1: Table S1), while some pro-inflammatory markers were downregulated in this group (*IL1RAP)* (Additional file 1: Table S1), suggesting that cDDGS may have anti-inflammatory properties. On the other hand, *C5AR1*, which was also upregulated after the addition of cDDGS, is connected to inflammation. Moreover, the inhibition of *C5AR1* is proposed as a therapeutic strategy in cardiovascular diseases; nonetheless, the role of *C5AR1* in obesity and inflammation is still not fully understood. It was recently shown that *C5AR1* plays an important role in cardiac regeneration [39] and B-1 cell homeostasis [40]. Additionally, some researchers observed downregulation of *C5AR1* expression in adipose tissue with increasing body mass index in women [41], while other researchers reported upregulation of *C5AR1* expression in acquired adiposity in monozygotic twins [42]. Our results suggest a strong connection between diet and macrophage polarization and adipose inflammation, but we are not able to clearly state its consequences for health. Therefore, further in vitro investigations are needed to fully evaluate the effects of the bioactive ingredients of cDDGS on these processes in adipose tissue. The same finding applies to IgE receptor activation *(FCER1G, MS4A2),* which is mainly triggered by the presence of parasites or bacteria and connected to allergy response.

In the case of cDDGS, the factor that may activate the complement system may be beta-glucan, which is an element of dietary fiber of grain origin that can be present also in yeast cell walls. Upregulation of inflammation and immunity genes was observed in pigs fed a high-fat high-fiber diet [15]. Researchers concluded that dietary fat and fiber may have an antagonistic effect on proinflammatory and anti-inflammatory signaling pathways. However, contrary to our results, those researchers observed immune gene modulation only in perirenal fat but not in subcutaneous fat. It is possible that cDDGS may have much stronger effects than fiber from straw, leading to gene expression modulation also in subcutaneous fat. On the other hand, such a reaction may be the result of the presence of mycotoxins in cDDGS. Biochemical analysis revealed no such contamination in our feed; however, due to the wide range of feed contaminants, it cannot be completely ruled out.

The limitation of our study is that our observation has not been proven using in vitro cultured porcine adipocytes; however, this genome-wide experiment confirmed several previous reports on in vitro and in silico interactions between regulatory genes of fatty acid biosynthesis and genes coding for enzymes engaged in this process in human and murine cells [16, 17]. Including both sexes in the experiment could also be considered a limitation; however, to assess possible bias, we performed DESeq2 analysis of 6 samples from group I vs 6 samples from group II with balanced sexes and found that the differences are negligible. The key identified genes are among statistically significant genes in both analysis (Additional file 4: Table S4). Moreover functional analysis with String software showed that genes engaged in discussed pathways (fatty acid biosynthesis, oxidative phosphorylation and complement and coagulation cascade) pathways are overrepresented in this dataset (Additional file 4: Table S4). Some doubts may arise from the fact that the diets containing cDDGS had a slightly higher fat content than the other diets, but the difference was only ~ 4%, which is not enough to individually induce such changes in the transcriptome.

## Conclusions

Currently, obesity, cardiovascular and neurodegenerative diseases are some of the main causes of death in highly developed countries. This problem has also begun to include domestic animals such as dogs or cats, and cDDGS seems to be a product that could counteract these disorders. The results of our research show that the addition of cDDGS to the diet causes a reduction in the expression of genes involved in lipogenesis and cellular respiratory processes while stimulating the genes of the immune system. The use of this product in livestock diets for many years shows the relative safety of cDDGS; however, further studies are required on laboratory animals and in in vitro cultures to fully understand the molecular mechanisms that are activated after the addition of cDDGS. Moreover, isolating biologically active components of cDDGS and exploring their activity would be useful in assessing the potential of cDDGS as a health-promoting component of the diet.

## Materials and methods

### Animals and diets

All procedures included in this study relating to the use of live animals were in agreement with the local Ethics Committee for Experiments with Animals in Cracow (Resolution No 912, of the day 26.04.2012).

The experiment was performed at the pig farm of the Experimental Station of the National Research Institute of Animal Production on the pigs born there. National Research Institute of Animal Production was the owner of all animals used in the study. Experimental procedures regarding the nutrition, housing and management of the animals are described elsewhere [6, 7]. Briefly, 32 crossbred fatteners originating from sows (Polish Landrace × White Large Polish) mated with a boar (Duroc × Pietrain) were kept in individual straw-bedded pens in uniform conditions. The animals were healthy and as similar in regard to weight as possible. The animals were divided into four dietary groups, eight pigs in each group (four gilts and four barrows). The diets of all groups were isonitrogenous and isoenergetic and were formulated to cover the nutritional requirements of the pigs. The ingredient composition and nutritive value of the diets, as well as the fatty acid compositions of the fat sources and feed mixtures, are presented elsewhere [6]. Briefly, the diets differed among each other in terms of the presence of 20% cDDGS and a source of fat: group I = -cDDGS+ 3% rapeseed oil, group II = +cDDGS+ 3% rapeseed oil, group III = +cDDGS+ 3% beef tallow, and group IV = +cDDGS+ 3% coconut oil. The control diet in group I was characterized by a slightly lower amount of crude fat (13.7% vs 16.5–18.4%), a lower amount of fiber and a slightly higher amount of starch (45.4% vs 38.9–39.5%) compared to the diet in the other groups, while the dietary content of crude protein was similar among all groups (Table 5). Experimental fattening lasted from 60 to 118 kg of live animal weight (approximately two months).. At the end of the experiment, the pigs were transferred to a professional slaughterhouse, where they were slaughtered in accordance with the legal regulations in force at the commercial slaughterhouse. No other pharmacological anesthesia and euthanasia methods were used. The experiment was planned for the sequencing of 24 libraries, and the 7 samples most uniform in regard to weight from each dietary group of backfat were collected for transcriptome analysis (one extra for each group) and stored in a freezer (− 85 °C). The meat from the experimental animals was intended for consumption, since the dietary treatments did not contain any harmful components.

**Table 5:** Nutritional values and fatty acids composition (g/100 g of detected fatty acids) of feed mixtures used in the experiment

Item	Group I (-cDDGS +rapeseed oil)	Group II (+cDDGS +rapeseed oil)	Group III (+cDDGS +beef tallow)	Group IV (+cDDGS +coconut oil)
Metabolizable energy (MJ)	13.5	13.8	13.6	13.8
Crude protein (g)	165.0	162.0	161.0	161.0
Crude fiber (g)	38.5	42.3	42.3	42.3
Crude ash (g)	45.2	42.5	42.6	42.6
N-free extractives (g)	581.7	568.4	568.7	568.7
Crude fat(g)	49.6	68.8	66.5	68.5
Crude fat, % of total ME	13.7	18.4	16.5	18.1
Starch (g)	425.0	372.0	372.0	372.0
Starch, % of total ME	45.4	38.9	39.5	39.0
C8:0	0.13	0.07	0.13	3.81
C10:0	–	–	0.19	3.53
C12:0	0.50	0.26	0.76	25.1
C14:0	0.25	0.25	0.95	6.85
C16:0	12.4	11.0	19.8	11.6
C16:1	0.20	0.18	0.86	0.18
C18:0	2.35	2.56	8.96	2.50
C18:1	43.0	35.5	31.2	15.20
C18:2	30.3	41.1	31.9	27.7
C18:3	5.27	5.28	2.72	1.97
γC18:3	0.06	–	0.02	–
C20:0	0.96	0.81	0.53	0.36
C20:4	0.04	0.03	0.09	0.02
C20:5	0.14	0.10	0.05	0.05
C22:0	0.71	0.50	0.22	0.19
C22:1	1.22	0.77	0.43	0.33
C22:6	0.62	0.43	0.21	0.15
SFA^a^	17.35	15.5	31.6	53.9
UFA^a^	80.9	83.4	67.5	45.6
MUFA^a^	44.4	36.4	32.4	15.7
PUFA n-6^a^	30.4	41.1	32.1	27.7
PUFA n-3^a^	6.04	5.80	2.98	2.16
PUFA^a^	36.4	46.9	35.1	29.9
SFA/UFA ratio	0.21	0.19	0.47	1.18
IV, g/100g^b^	104.5	116.2	90.5	66.6

^a^Sum of fatty acids: saturated (SFA), unsaturated (UFA), monounsaturated (MUFA) polyunsaturated (PUFA)

^b^IV—iodine value of fat

### RNA isolation, RNA-seq and qPCR procedures

Total RNA was isolated using the Direct-zol RNA kit (Zymo Research, Irvine, CA) according to the manufacturer’s protocol. The quality and concentration of the obtained RNA were evaluated on a TapeStation Instrument (Agilent Technologies, Inc., Santa Clara, CA). Since we could not obtain undegraded RNA from our samples (probably due to the problems with the freezer) we decided to employ the SMARTer Stranded Total RNA Sample Prep Kit - HI Mammalian (Takara, Clontech, Mountain View, CA) dedicated for library construction from total RNA with a RIN (RNA integrity number) between 3 and 10. We obtained satisfactory RNA from 23 samples (Additional file 5: Table S3). A 260 /280 ratio was between 1.98 and 2.06. The entire protocol included several steps: ribosomal RNA depletion with RiboGone technology, first-strand cDNA synthesis and purification, Illumina-specific library amplification by PCR, and RNA-Seq library purification. The quality and concentration of the libraries were evaluated on a TapeStation 2000 Instrument (Agilent Technologies, Inc., Santa Clara, CA) and a Qubit fluorometer (Thermo Fisher Scientific, Waltham, MA). RNA libraries (*n* = 23 [5–7 per group]) were prepared for sequencing using standard Illumina protocols: the libraries were diluted to a final concentration of 10 nM, and 5 or 6 samples were pooled. Cluster generation was performed on a cBot instrument (Illumina, Inc., San Diego, CA) using the TruSeq SR Cluster Kit v3-cBot-HS. Sequencing (single-end) was performed in two replicates on an Illumina HiScanSQ 2000 in one flow cell with the TruSeq SBS Kit v3 - HS (50 cycles).

Validation of the RNA-seq results was performed for 8 genes *(ACACA, ACLY, FASN, ALAS1, FITM2, C5AR1, VSIG4, MS4A2*) by quantitative real-time PCR (qPCR). The cDNA was synthesized with the cDNA Archive Kit (Thermo Fisher Scientific, Waltham, MA). The qPCR was performed in duplicate on a QuantStudio 7 Flex instrument (Thermo Fisher Scientific, Waltham, MA) under the fast thermal profile. The reaction mix contained 1 μl of cDNA, 5 μl of GoTaq® qPCR Master Mix (Promega Corporation, Madison, WI), 0.1 μl of CXR dye, 3.23 μl of water, 0.17 μl of 60 × TaqMan assay for *OAZ1* (endogenous control) amplification (*Assay ID: Ss03397505_u1*) and 0.5 μl of 20 × TaqMan gene expression assay for amplification of the target gene (*ACACA assay ID: Ss03389963_m1, ACLY assay ID: Ss03389566_m1, FASN assay ID: Ss03386194_u1, ALAS1 assay ID: Ss04652684_m1, FITM2 assay ID: Ss03267236_m1, MS4A2 assay ID: Ss03394007_m1, VSIG4 assay ID: Ss04328828_g1,* and *C5AR1 assay ID: Ss03375530_u1).* The relative quantitation (RQ) of each sample was calculated basing on the ΔΔCt method using QuantStudio Real-Time PCR software.

### RNA-Seq data processing and statistical methods

The demultiplexing of the RNA-seq samples was performed with the bcl2fastq conversion software v1.8.4 (Illumina). The quality check, trimming of reads and mapping of reads were conducted with FastQC, FLEXBAR and TopHat software, respectively. The mapping statistics and read counts were generated with SAMStat, RSeQC, RSEM and HTSeq software. Differential expression analysis was performed using DEseq2 software. Genes with an adjusted p of < 0.05 and a fold change > ± 1.3 were regarded as differentially expressed. We decided to apply a relatively low fold-change threshold since we expected rather subtle differences in gene expression. Classification of differentially expressed genes (DEGs) was performed with the PANTHER Classification System (Bonferroni’s correction was applied, http://pantherdb.org/), and further analyses were conducted with the Reactome database (http://www.reactome.org/). To compare the RNA-seq results with the qPCR results, the Pearson correlation between the fold change obtained after RNA-seq and qPCR was calculated using SAS software. Statistical differences between phenotypic traits between the groups were evaluated using ANOVA (Duncan’s multiple range test), and for qPCR data using nonparametric ANOVA (Kruskal-Wallis test) and GLM with covariates of sex and diet to assess the significance of each factor. (SAS Enterprise guide 7.1).

## Additional files

Additional file 1: **Table S1.** Results of the identification of DEGs by DESeq 2 software of all comparisons. (XLSX 98 kb)

Additional file 2: **Table S2.** List of all enrichments identified by String software among DEGs in (-cDDGS+rapessed oil) vs (+cDDGS+rapeseed oil) comparison. (XLSX 58 kb)

Additional file 3: **Figure S1.** Validation of RNA-seq results by qPCR.– comparison of fold changes between group I (*n* = 7) vs group II + III + IV (*n* = 16) obtained after RNA-seq and qPCR. (JPG 32 kb)

Additional file 4: **Table S4.** Results of the identification of DEGs between groups I and II by DESeq 2 software and functional analysis with String software of identified DEGs after excluding one sample to obtain sex balance among groups. (XLSX 6539 kb)

Additional file 5: **Table S3.** Characteristics of samples used in the study. (XLSX 10 kb)

## Abbreviations

*AACS*: Acetoacetyl-CoA synthetase
*ACACA*: Acetyl-CoA carboxylase alpha
*ACC*: Acetyl-CoA carboxylase
*ACLY*: ATP citrate lyase
*ALAS1*: 5′-Aminolevulinate synthase 1
*ARVCF*: ARVCF, delta catenin family member
*C1QTNF6*: C1q and tumor necrosis factor related protein 6
*C1QTNF9*: C1q and tumor necrosis factor related protein 9
*C5AR1*: Complement component 5a receptor 1
*CASR*: Calcium sensing receptor
*CEP128*: Centrosomal protein 128
*ChREBP*: Carbohydrate-responsive element-binding protein
*COX7A2*: Cytochrome c oxidase subunit VIIa
*COX8A*: Cytochrome c oxidase subunit VIIIA
*CYB5B*: Cytochrome B5 type B (outer mitochondrial membrane)
*CYGB*: Cytoglobin
*CYP2U1*: Cytochrome P450 family 2 subfamily U member 1
*DSPP*: Dentin sialophosphoprotein
*F13A1*: Coagulation factor XIII, A1 polypeptide
*F3*: Coagulation Factor III (thromboplastin, tissue factor)
*FAM105A*: Family with sequence similarity 105, member A
*FASN*: Fatty acid synthase
*FCER1G*: Fc fragment of IgE, high affinity I, receptor for; gamma polypeptide
*FITM2*: Fat storage-inducing transmembrane protein 2
*GFM1*: G elongation factor mitochondrial 1
*GIMAP4*: GTPase, IMAP family member 4
*HIP1R*: Huntingtin interacting protein 1 related
*HMGCS1*: 3-hydroxy-3-methylglutaryl-CoA synthase 1
*KAT8*: Lysine acetyltransferase 8
*MS4A2*: Membrane spanning 4-domains A2
*MUC2*: Mucin 2
*MYO1E*: Myosin IE
*NCOA7*: Nuclear receptor coactivator 7
*NDUFA10*: NADH dehydrogenase [ubiquinone] 1 alpha subcomplex subunit 10
*NDUFB8*: NADH:ubiquinone oxidoreductase subunit B8
*NDUFS5*: NADH:ubiquinone oxidoreductase subunit S5
*PCDH12*: Protocadherin 12
*PDHX*: Pyruvate dehydrogenase complex component X
*PEPT1*: Peptide transporter 1
*RGS1*: Regulator of G protein signaling 1
*RTN3*: Reticulon 3
*SCD*: stearoyl-CoA desaturase (delta-9-desaturase)
*SLC25A1*: Solute carrier family 25 member 1
*SP110*: SP110 nuclear body protein
*SPARCL1*: SPARC like 1
*TAF4B*: TATA-box binding protein associated factor 4b
*TAS1R3*: Taste 1 receptor member 3
*TCHP*: Trichoplein keratin filament binding
*UMPS*: Uridine monophosphate synthetase
*UQCRFS1*: Ubiquinol-cytochrome c reductase, rieske iron-sulfur polypeptide 1
*UQCRQ*: Ubiquinol-cytochrome c reductase, complex III subunit VII
*VSIG4*: V-set and immunoglobulin domain containing 4
*ZEB1*: zinc finger E-box binding homeobox 1
*ZMAT1*: Zinc finger matrin-type 1
