# Supplementary figures and images for: Corn dried distillers grains with solubles (cDDGS) in the diet of pigs change the expression of adipose genes that are potential therapeutic targets in metabolic and cardiovascular diseases
Source: BMC Genomics. 2018 Dec 3;19:864. doi: 10.1186/s12864-018-5265-x (PMC6276254; doi:10.1186/s12864-018-5265-x)

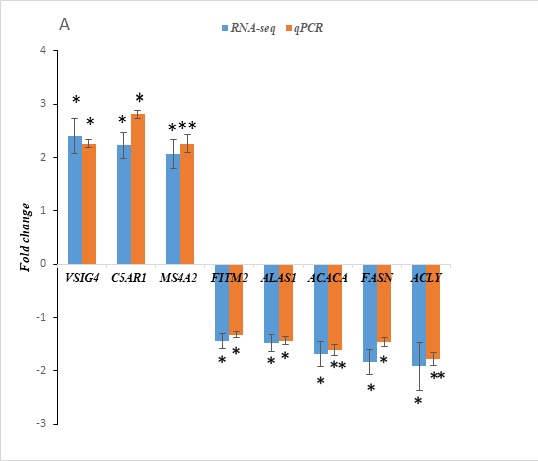

Supplement: Supplementary file 3 — Figure S1. Validation of RNA-seq results by qPCR.– comparison of fold changes between group I (n = 7) vs group II + III + IV (n = 16) obtained after RNA-seq and qPCR. (JPG 32 kb) [file 12864_2018_5265_MOESM3_ESM.jpg]
